# Early Lift of Restrictions in Mongolia During the COVID-19 Pandemic: Child Rights, Public Trust, and Social Inequality

**DOI:** 10.1177/10105395221108592

**Published:** 2022-06-25

**Authors:** Javkhlanbayar Dorjdagva, Enkhjargal Batbaatar, Jussi Kauhanen

**Affiliations:** 1 Institute of Public Health and Clinical Nutrition, Faculty of Health Sciences, University of Eastern Finland, Kuopio, Finland; 2 Department of Social Sciences, Faculty of Social Sciences and Business Studies, University of Eastern Finland, Kuopio, Finland

Even though safe and effective COVID-19 vaccines are in use all around the world,^1^ the pandemic is far from over. Global vaccine inequities and the emerging COVID-19 variants are just two of the reasons for concern.^2^ In these circumstances, the role of non-medical countermeasures is essential to allow health systems to cope with the burden of COVID-19.

Mongolia started its coronavirus vaccination rollout in early 2021, and by the end of that year, 66.5% of the total population had been fully vaccinated with 28.0% of them having received a booster shot.^3^ Despite the successful vaccination campaign, the number of cases and deaths has been relatively high. As of March 10, 2022, there were a total of 914,536 COVID-19 cases (about 27 out of every 100 people), and the total COVID-19 deaths amounted to 2105.^4^ This may be partially explained by the fact that some restrictions were lifted prematurely. In this paper, we focused on seven unintended consequences in the Mongolian context that could have resulted from the easing of non-medical countermeasures prematurely and instead relying mainly on the vaccination campaign during the pandemic.

First, COVID-19 cases and deaths. On February 23, 2021, as the vaccination campaign started, the government lifted restrictions in an attempt to promote an economic recovery.^5^ On that day, a total of 30 new confirmed cases were registered, with a total accumulated number of 2723. Accordingly, by April 10, the number of new cases reached 689; of these slightly under 10% were registered in rural areas.^3^ The government reacted by placing the country under lockdown from April 10 to 25. As a result, the number of new cases declined. However, the presidential election campaign was held throughout Mongolia, and some mass gatherings did take place. In July, the restrictions on movement were gradually lifted and domestic tourism increased steeply (Figure 1). Consequently, Mongolia became a COVID-19 hotspot. On September 7, the highest number of daily new cases, 3963, was reported but now almost two-thirds of these cases were in rural areas,^3^ where the capacity of the health system to deal with a pandemic is generally poor. From September to December 2021, the COVID-19 situation in Mongolia improved as the number of daily new cases declined consistently. Nevertheless, as the New Year approached, the country faced a clear risk of a COVID-19 resurgence as it is a tradition in Mongolia that all organizations celebrate the New Year by gathering in restaurants or bars. The government restricted public organizations to celebrate the holiday as a preventive measure; however, did allow private organizations to celebrate. As a result, the number of daily new cases increased sharply from 34 to 3,282 between December 13, 2021, and January 21, 2022.^3^ This highlights the argument that the decision to lift restrictions should have been made on an epidemiological basis with respect to both local and national situations within Mongolia.

**Figure 1.**
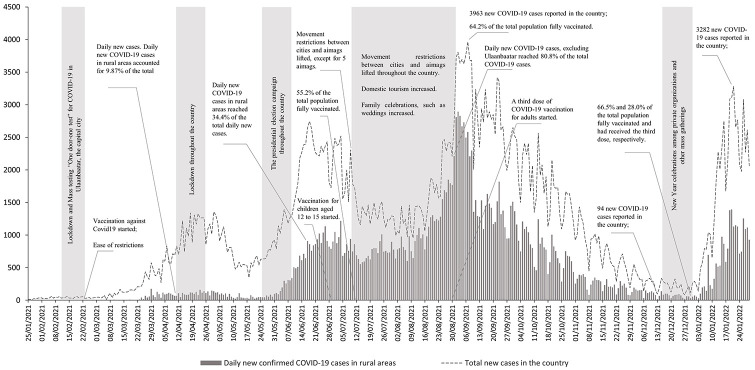
Daily new confirmed COVID-19 cases in Mongolia. Source: Data were adapted from the Ministry of Health.^3^

Second, the trade-off between economic recovery and saving lives during the pandemic. We argued that the inappropriate timing when restrictions were eased had been associated with substantial economic and social costs due to increased caseload and number of deaths, and in fact, these costs might be much higher than the potential economic benefits gained from the early reopening of the Mongolian economy. It is evident that a sustainable economic recovery during and after the pandemic is not possible without first saving lives.

Third, social inequality. There have been unacceptable social inequalities in exposure and vulnerability to coronavirus. The likelihood of catching the disease and the possibility of suffering severe or fatal outcomes are affected by many medical and non-medical factors, for example, crowded and poor housing, and the presence of pre-existing illnesses.^6^ Therefore, poor and vulnerable groups in Mongolia had faced a greater risk of the negative consequences that followed from the decision to lift the restrictions in the midst of a worsening pandemic. This, in turn, may lead to even wider social inequalities.

Fourth, non-COVID-19 deaths. It has been shown that lifting restrictions prematurely during this pandemic was likely to cause a rebound in COVID-19 cases and thus to rapidly increase the demand for health care.^7^ This burdened the healthcare system’s limited resources, such as the number of healthcare workers, and affected the proper management of other diseases. Ultimately, increases in the non-COVID-19 mortality may have occurred in the population.

Fifth, public trust. New mutations of the virus and breakthrough COVID-19 infections in vaccinated individuals have become critical concerns globally.^8^ If COVID-19 measures are not tightened in a timely manner, there is a high risk of a disease surge not only among those who are unvaccinated but also an increased risk of breakthrough infections among those who have been vaccinated. Governmental inertia in this type of situation can critically harm the fight against the pandemic by diminishing public trust in the effectiveness of COVID-19 vaccines.

Sixth, children’s rights. Loosening restrictions prematurely due to high vaccination rates among adults is a dubious moral decision since it leaves unvaccinated children at risk and thus could violate the rights of children. Hence, proper restrictions to contain the disease are not only a public health and/or economic issue, but also a moral responsibility.

Seventh, air pollution and winter. Air pollution significantly increases the COVID-19 severity and associated mortality risk.^9^ An early easing of restrictions during the coldest winter months may well have created additional risks, especially in Mongolia where both outdoor and indoor air pollution are a major public health problem.^10^ Social events and mass gatherings inevitably facilitate the transmission of coronavirus, as people are packed together in locations where there is poor indoor air quality due to the seasonal cold temperatures.

In Mongolia, inarguably the vaccination campaign has been one of the successful measures taken during the pandemic. Nonetheless, the effectiveness of the policy would have been much better if restrictions had not been lifted prematurely during the early phases of the vaccination program. Effective coordination of all policies is needed from the Government to end this pandemic and prepare the country for future ones.

## References

[1] World Health Organization. Coronavirus disease (COVID-19): vaccines. Published 2022. Accessed December 4, 2021. https://www.who.int/news-room/questions-and-answers/item/coronavirus-disease-(covid-19)-vaccines.

[2] Ghebreyesus TA. Five steps to solving the vaccine inequity crisis. PLoS Glob Public Health. 2021;1(10):e0000032. doi:10.1371/journal.pgph.0000032. PMC10022218 36962118

[3] Ministry of Health of Mongolia. Coronavirus disease (COVID-19) situation report. Date unknown. https://covid19.mohs.mn/p/cat/post/57/.

[4] World Health Organization. COVID-19 situation in WHO—Western Pacific Region. Date unknown. Accessed March 22, 2022. https://experience.arcgis.com/experience/e1a2a65fe0ef4b5ea621b232c23618d5.

[5] Government of Mongolia. Mongolia has started vaccination for covid-19. Accessed November 23, 2021. https://mongolia.gov.mn/news/view/25753.

[6] Whitehead M Taylor-Robinson D Barr B. Poverty, health, and covid-19. BMJ. 2021;372:n376. doi:10.1136/bmj.n376. 33579719

[7] Ferguson N Laydon D Nedjati Gilani G , et al. Impact of non-pharmaceutical interventions (NPIs) to reduce COVID-19 mortality and healthcare demand. doi:10.25561/77482. PMC7140590 32270376

[8] Burki TK. Omicron variant and booster COVID-19 vaccines. Lancet Respir Med. 2022;10:e17. doi:10.1016/S2213-2600(21)00559-2. PMC8683118 34929158

[9] Wu X Nethery RC Sabath MB Braun D Dominici F. Air pollution and COVID-19 mortality in the United States: strengths and limitations of an ecological regression analysis. Sci Adv. 2020;6(45):eabd4049. doi:10.1126/sciadv.abd4049. PMC7673673 33148655

[10] Ganbat G Soyol-Erdene TO Jadamba B. Recent improvement in particulate matter (PM) pollution in Ulaanbaatar, Mongolia. Aerosol Air Qual Res. 2020;20:2280-2288. doi:10.4209/aaqr.2020.04.0170.

